# Rheological and Mechanical Properties of Thermoresponsive Methylcellulose/Calcium Phosphate-Based Injectable Bone Substitutes

**DOI:** 10.3390/ma11040604

**Published:** 2018-04-14

**Authors:** Öznur Demir Oğuz, Duygu Ege

**Affiliations:** Institute of Biomedical Engineering, Boğaziçi University, Rasathane St., Kandilli, 34684 İstanbul, Turkey; oznur.demir@boun.edu.tr

**Keywords:** methylcellulose, injectable bone substitutes, calcium phosphate cement, calcium sulfate, citric acid, gelatin, bone, rheological studies, injectability, mechanical properties

## Abstract

In this study, a novel injectable bone substitute (IBS) was prepared by incorporating a bioceramic powder in a polymeric solution comprising of methylcellulose (MC), gelatin and citric acid. Methylcellulose was utilized as the polymeric matrix due to its thermoresponsive properties and biocompatibility. 2.5 wt % gelatin and 3 wt % citric acid were added to the MC to adjust the rheological properties of the prepared IBS. Then, 0, 20, 30 and 50 wt % of the bioceramic component comprising tetracalcium phosphate/hydroxyapatite (TTCP/HA), dicalcium phosphate dehydrate (DCPD) and calcium sulfate dehydrate (CSD) were added into the prepared polymeric component. The prepared IBS samples had a chewing gum-like consistency. IBS samples were investigated in terms of their chemical structure, rheological characteristics, and mechanical properties. After that, in vitro degradation studies were carried out by measurement of pH and % remaining weight. Viscoelastic characteristics of the samples indicated that all of the prepared IBS were injectable and they hardened at approximately 37 °C. Moreover, with increasing wt % of the bioceramic component, the degradation rate of the samples significantly reduced and the mechanical properties were improved. Therefore, the experimental results indicated that the P50 mix may be a promising candidates to fill bone defects and assist bone recovery for non-load bearing applications.

## 1. Introduction

Since the late 1980s, there is ongoing research on the development of injectable bone substitutes (IBS) [1,2,3]. The most commonly studied IBS’s in dentistry and orthopedic applications are calcium phosphate cements (CPC) due to their excellent physical, mechanical and biological properties [3,4,5]. Since CPCs are biocompatible, bioactive, biodegradable and osteoconductive, there is tremendous research on their further development. CPCs are injectable, and they harden in vivo, after taking the shape of the defect site. Two of the most commonly used bioceramics for the production of CPCs are dicalcium phosphate dihydrate (DCPD, CaHPO_4_.2H_2_O) and tetra-calcium phosphate (TTCP, Ca_4_(PO_4_)_2_O) [4,6,7]. After a mixture of TTCP and DCPD is injected into the defect site, it transforms into hydroxyapatite (HA) [6,7,8,9]. Pure CPC bone substitutes have many drawbacks; such as the possibility of collapse under physiological conditions, poor degradability, as well as weak torsional and bending strength [10,11,12]. The addition of calcium sulfates (CSD) to CPCs may overcome some of these drawbacks, including the improvement of their injectability, mechanical strength, degradability, and osteointegration. CSD form after mixing α-calcium sulfate hemihydrate with distilled water. The addition of CSD into CPC leads to improvement of plasticity and the compression strength of the CPC [11,13,14]. 

To adjust the rheological properties, including injectability, setting temperature, mechanical properties of IBS, CPC and CSD mixture may be incorporated into a polymeric matrix. The presence of a polymeric matrix may also assist the permeation of body fluids into bone substitutes; therefore promoting three-dimensional cell migration, cell growth and ultimately ossification [4,15]. Cellulose is a promising polymer for this purpose; however, it is not water-soluble due to the presence of intra-molecular hydrogen bonding; which restricts its range of biomedical applications [16,17,18]. Therefore, hydrophilic and water-soluble derivatives of cellulose have emerged [19]. Methylcellulose (MC), which is one of the cellulose ether derivatives, has been extensively studied for biomedical applications. It also shows the potential for production of IBS due to its thermoresponsive properties [16]. Research shows that MC enhances injectability, mechanical properties, and osteoconductivity of CPC-based IBS [20,21,22,23]. In their study, Liu et al. used 8, 10 and 12 wt % of MC to prepare injectable, biocompatible and biodegradable hydrogels. Overall, 10 wt % MC led to the most suitable rheological properties for biomedical applications including viscosity, setting time and storage modulus [24]. 

In addition to that, like MC, gelatin has been added to CPC pastes; which does not only mimics the organic matrix of bone, but also contributes to the hardening of CPC and improves the workability of cement pastes [25,26]. Moreover, the addition of gelatin into MC enables gelation at the physiological temperature and also provides improved cell-surface interactions due to the presence of its arginine-glycine-aspartate or RGD group [26,27,28]. Nishinari et al. [29] observed that the interaction between the non-substituted hydroxyl group in methylcellulose and the carboxyl group in gelatin reduces the gelling temperature of MC. [9,30]. In many studies, 2–3 wt % gelatin was added in hydrogels to improve their biocompatibility. Additionally, gelatin addition improved the mechanical properties of hydrogels [3,31,32].

Experimental studies show that the addition of different salts to MC solution also reduces the gelling temperature to the physiological temperature [16,19,33]. Citric acid is a cost-effective, non-toxic additive which optimizes the physical properties of IBS; such as degree of swelling, setting time, viscosity and mechanical properties [9,30]. The carboxylate group in citric acid reacts with the hydroxyl groups of MC and the amino groups of gelatin via esterification. Citric acid acts as a liquefier and therefore also enhances the injectability and workability of CPC pastes [34,35]. It also improves the strength of CPC pastes due to its high salting-out effect according to the Hofmeister series [9]. In previous studies, citric acid was incorporated in calcium phosphate cements up to 3 wt%. With an increase of wt % of citric acid, setting time and injectability decreased which was described as acceptable for clinical applications [36].

In the current study, IBS was prepared by mixing MC, gelatin, citric acid, and different wt % of bioceramic comprising of CPC and CSD. As previous studies indicated improved physical properties for use of 10 wt % MC, 3 wt % citric acid and 2.5 wt % gelatin in prepared scaffolds; in this study, these values were kept constant. With this novel combination of polymers and CPC, an ideal IBS for orthopedic applications can be developed which stimulates new bone formation. 20, 30, and 50 wt % of CPC/CSD–based bioceramic powder was added in the polymeric matrix to produce IBS. The chemical structure of the samples was investigated using X-ray Diffraction (XRD). The physical handling and mechanical properties of the IBS samples were analyzed with rheological studies, compressive strength measurements and injectability tests. After that, the in vitro degradation studies were carried out. Overall, the experimental studies suggest that MC/bioceramic powder-based IBS were promising in terms of their rheological, mechanical and degradation properties for future bone treatment applications. 

## 2. Materials and Methods

### 2.1. Materials

Methylcellulose (MC, viscosity 15 cps, Mw 14 kDa, DS 1.5–1.9), gelatin (from bovine skin, gel strength ~225 g Bloom, Type B), sodium citrate tribasic dihydrate (SC, C_6_H_5_Na_3_O_7_ Mw 294.10 g/mol, mp: >300 °C (lit.), pH 7.0–9.0 at 25 °C), calcium hydrogen phosphate dihydrate (DCPD, CaHPO_4_.2H_2_O, Mw 172.09 g/mol, d:2.31 g/mL), monetite (CaHPO_4_, Mw 136.06 g/mol), calcium carbonate (CaCO_3_, Mw 100.09 g/mol, d: 2.93 g, ≤30 µm particle size) and phosphate buffer saline (PBS, tablets, pH 7.4 at 25 °C) were purchased from Sigma Aldrich (Sigma Aldrich, Taufkirchen, Germany). Calcium sulfate dihydrate (CSD, CaSO_4_·2H_2_O, Mw 172.17 g/mol) was purchased from Merck (Merck KGaA, Darmstadt, Germany). 

### 2.2. Preparation of Injectable Bone Substitutes (IBS) Samples

#### 2.2.1. Preparation of the Polymeric Solution

Methylcellulose (MC) solution was prepared by dissolving 6 g of MC powder in distilled water at 90 °C until MC’s complete dissolution. The prepared solution was stored at 4 °C overnight to obtain a clear MC solution [33]. 1.875 g of gelatin was dissolved in 12.5 mL of distilled water at 50 °C and was allowed to cool down before use [24]. 2.25 g of sodium citrate dihydrates (SC) solution was prepared by dissolving SC salts in 12.5 mL distilled water at room temperature [19]. Then all solutions were blended to a final concentration of 8.0, 2.5, and 3.0 wt % of MC, gelatin, and SC, respectively.

#### 2.2.2. Preparation of the Bioceramic Powder Mixture

The bioceramic component of IBS samples consist of TTCP/HA-based powder, DCPD, and CSD. Analytical grade DCPD and CSD were used without any further purification. TTCP/HA-based powder was synthesized by heating an equi-molar mixture of monetite (CaHPO_4_) and calcium carbonate (CaCO_3_) at 1500 °C for 6 h, using a 5 °C/min heating rate and 10 °C/min cooling rate [37]. CPC mixture was prepared by mixing an equi-molar mixture of DCPD and TTCP/HA-based powder and then this CPC mixture was added to the CSD in a 4 to 1 mass ratio [38]. 

After this, different wt % of the bioceramic powder phase was added to the polymeric component. Table 1 shows the compositions of prepared IBS samples. 

### 2.3. Characterization of IBS Samples

#### 2.3.1. XRD Analysis

XRD (Rigaku D/MAX-Ultima+/PC, Austin, TX, USA) analysis was performed to examine the synthesized powder and lyophilized IBS samples at 40 kV and 30 mA with a step size of 0.01° between 20° and 40° in a fixed time mode at Bogaziçi University, Istanbul, Turkey [33]. 

#### 2.3.2. Fourier Transform Infrared Spectroscopy (FTIR) Analysis

Functional groups of the synthesized powder were detected by using a Perkin Elmer FTIR spectrometer (Perkin Elmer, Waltham, MA, USA) from 4000 to 400 cm^−1^ using the KBr pellet technique at Yildiz Technical University, Istanbul, Turkey [39].

#### 2.3.3. Injectability Measurements

Injectability of the samples was qualitatively evaluated by extruding the IBS samples through a disposable syringe, using an 18-gauge needle, in PBS at 37 °C. Each syringe was filled with approximately 2 g of IBS, which was then extruded from the syringe manually at a constant speed [24,39].

#### 2.3.4. SEM Analysis

The morphology and the internal porous structure of the lyophilized IBS samples were observed by using Scanning Electron Microscopy (SEM) (Zeiss, Evo LS10, Oberkochen, Germany) with 10 kV accelerating voltage at Yildiz Technical University, Istanbul, Turkey [30]. Samples were coated with gold-palladium before the experiment.

#### 2.3.5. Rheological Measurements

Rheological measurements of IBS samples were performed by using a stress-controlled rheometer with a parallel plate geometry (diameter: 15 mm) (Anton Paar, MCR302, Graz, Austria) at Bogaziçi University, Istanbul, Turkey. The mechanical properties of the samples were measured at 0.1% strain and 10 rad/s frequency within the linear viscoelastic region of IBS. The oscillation amplitude and frequency sweep were carried out at 37 °C. A temperature sweep was performed from 15 to 45 °C at a heating rate of 2 °C/min in order to determine the gelation and setting temperatures. A time sweep was carried out at 37 °C to investigate the gelation kinetics. Finally, the shear thinning properties of IBS samples were analyzed both at 25 and 37 °C [24,40].

#### 2.3.6. Compressive Strength Measurements

IBS samples were molded into columns of 7.0 mm of diameter and 10.0 mm length. The compressive strength of the samples was measured using a Geratech SH-500 (Geratech, Taiwan) and SH-20 testing device (Geratech, Taiwan) at Bogaziçi University, Istanbul, Turkey. The compressive strength of IBS samples was taken at 15% strain. Measurements were taken on days 1,3, 5 and 7 and 14 in an atmosphere of 100% humidity at 37 °C (n = 5) [41]. 

#### 2.3.7. pH Changes

IBS samples were molded into discs, and after setting of the cement phase, they were immersed in PBS solution at 37 °C. The pH values of the PBS solution were measured at different time intervals including 5,10, 20, 30, 60, and 120 min, 1,3, 5, 7, 14, 21 and 28 days (n = 3) [41]. 

#### 2.3.8. In Vitro Degradation

The degradation behavior of the IBS samples was measured in PBS at 37 °C. IBS samples were immersed in 12 well plates. At pre-determined times, the IBS samples were lyophilized and weighed. The remnant dry weight was calculated using Equation (1) where W_0_ is the initial weight of the dry IBS samples and W_t_ is the dry weight of the IBS samples after t days of incubation [24].

(1) $$\%\;=\frac{W_{t}}{W_{0}}\times 100\left[\%\right]$$

## 3. Results and Discussion 

### 3.1. Analysis of the Synthesized Powder

**Figure 1:**
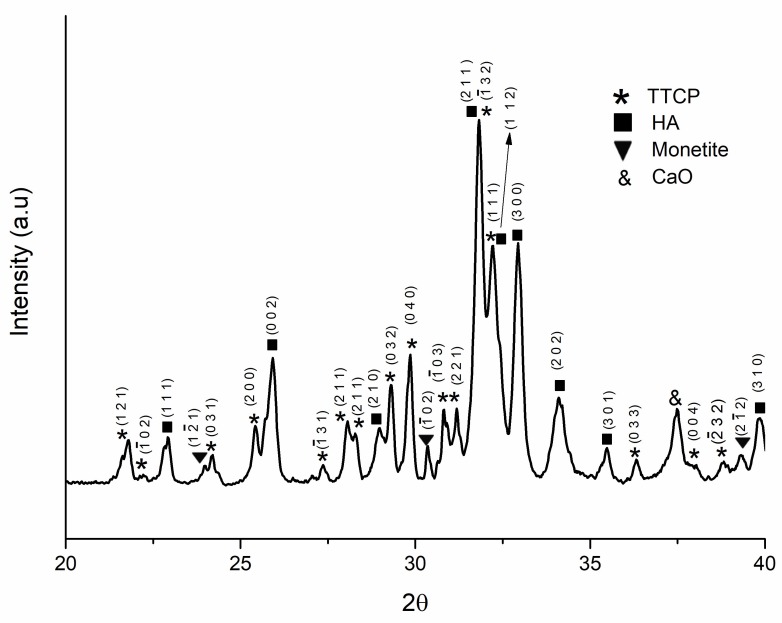
XRD (X-ray Diffraction) pattern of synthesized (tetracalcium phosphate/hydroxyapatite) TTCP/HA-based powder.

The TTCP/HA-based powder was synthesized as described in Section 2.2.2. Figure 1 shows the XRD analysis of the synthesized powder. The relevant Miller Indices of TTCP, HA and monetite are also presented.

Figure 1 shows that TTCP and hydroxyapatite peaks were detected since the powder was furnace-cooled [9,37,42,43]. A trace amount of monetite and calcium oxide (CaO) were also found from the XRD analysis. The matched peaks with JCPDS file No. 25-1137, No. 09-0432 and No. 09-0080 is given in Figure S1.

**Figure 2:**
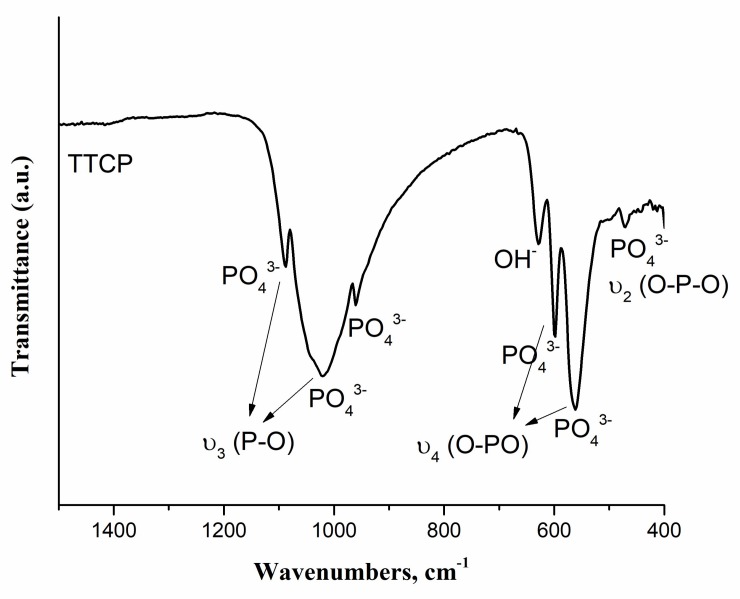
Fourier transform infrared (FTIR) spectrum of TTCP/HA-based powder by using KBr pellet method.

Figure 2 shows the FTIR spectrum of the synthesized powder.

In FTIR spectrum, absorption peaks were found at 452, 471, 502, 567, 600, 628, 958, 987, 1044, 1091, 1625, 1922, 2001, 2077, 3435, 3570 and 3643 cm^−1^. As labelled in Figure 2, PO_4_^3−^ bands of TTCP were detected in the spectrum which were found similar with that of literature [43,44,45]. OH^−^ stretching bands located at 628 and 3570 cm^−1^ indicated the presence of HA [42,46]. Absorption bands between 3000–3600 cm^−1^ indicated H_2_O adsorbed. The peaks located at 1625, 1922, 2000 and 2077 cm^−1^ indicated the presence of carbonate content. These peaks were possibly found due to carbon dioxide absorption from the atmosphere [47]. Therefore, FTIR results supported the results observed from XRD spectrum which indicated synthesis of TTCP/HA-based powder [37,44]. 

### 3.2. Injectability of IBS Samples

**Figure 3:**
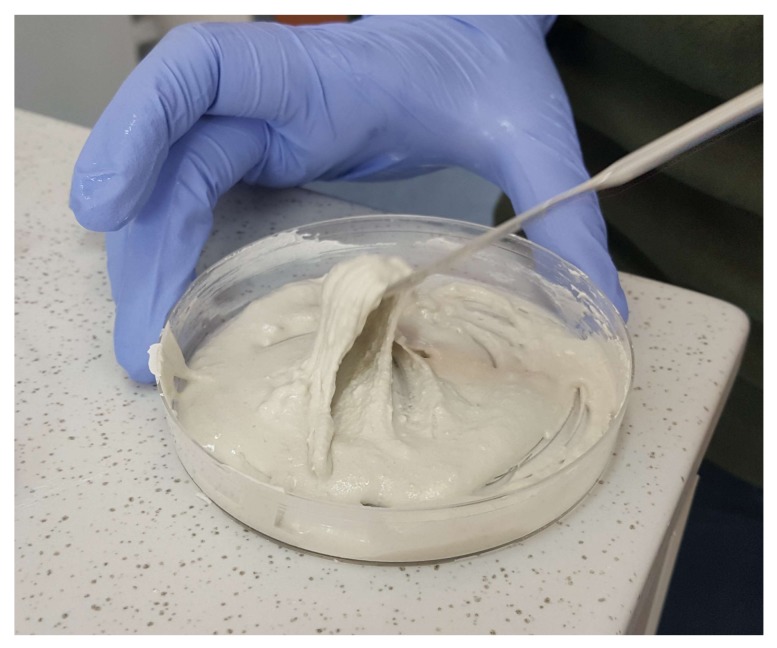
Chewing gum-like consistency of P50 samples after mixing of bioceramic and polymeric components.

Figure 3 shows that the mixture of bioceramic and polymeric components have a chewing gum-like consistency after mixing.

Since the IBS samples had a chewing gum-like consistency, IBS samples can be molded into the desired shape of the complex bone defects. This consistency was achieved as a result of the presence of the liquid phase [12]. Figure S2 shows that all of the IBS samples possess cohesive stability and moldability. According to the extrusion videos, P30 and P50 samples had a higher stability than P20, as P20 pastes had a tendency to disintegrate during the extrusion process. Figure S3 shows that all IBS samples had high degree of injectability. Higher wt % of bioceramic components were also introduced into the polymeric component; however, these samples could not be extruded through 18-gauge. Therefore, the maximum wt % of bioceramic component was set at 50%. 

### 3.3. Morphology of the IBS Samples

**Figure 4:**
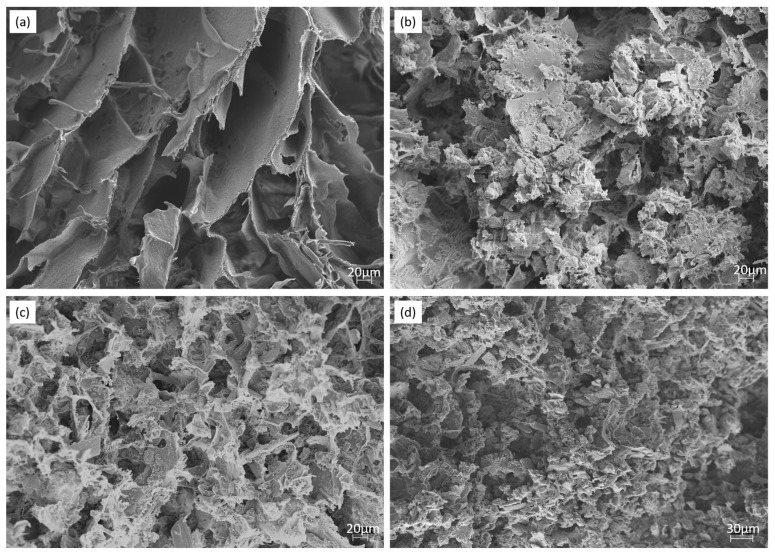
SEM images of (**a**) P0, (**b**) P20, (**c**) P30, (**d**) P50 IBS samples at a magnification of 500×.

Figure 4 shows the morphologies of lyophilized hydrogels studied by SEM. 

Figure 4 shows that all of the IBS samples had highly porous microstructure. When the wt % of the bioceramic component increased, the pore size decreased. P0 revealed that scaffolds had an interconnected, porous structure. SEM shows that bioceramics were well-adhered on the polymeric phase. Moreover, the bioceramic component was found to be homogenously distributed in the polymeric component [42,48] .

### 3.4. Rheological Measurements

**Figure 5:**
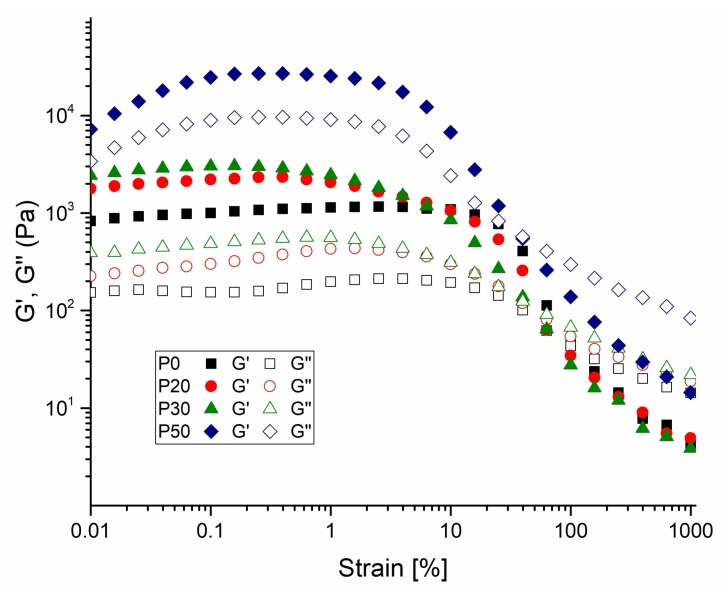
Amplitude-dependent variation of G’ and G″ changes of P0, P20, P30 and P50 IBS samples at a frequency of 10 rad/s at 37 °C (results expressed as mean ± standard error, n = 3).

Rheological measurements of IBS samples containing different wt % of the bioceramic component were evaluated. Figure 5 shows the amplitude sweep measurement of IBS samples. 

**Figure 6:**
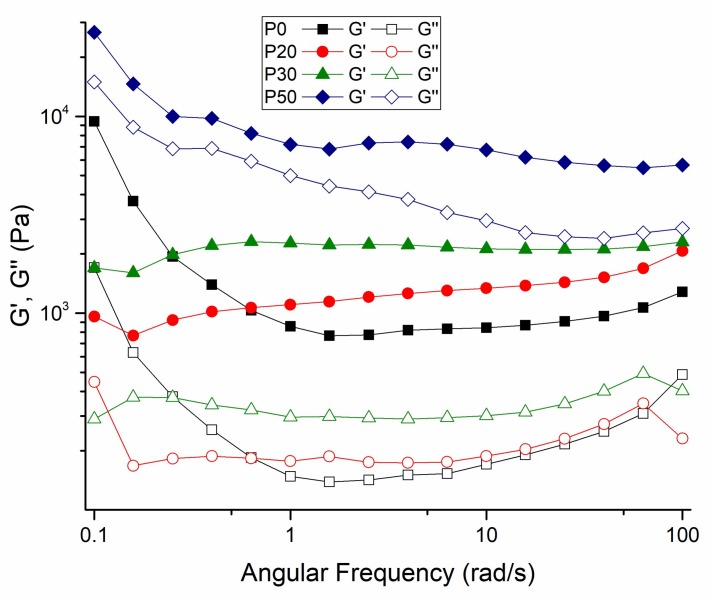
Frequency-dependent variation of G′ and G″ changes of P0, P20, P30 and P50 IBS samples at a 0.1% strain at 37 °C (results expressed as mean ± standard error, n = 3).

Compared with P0 samples, other IBS samples present a broader linear viscoelastic region at 37 °C; as the strain required to break the network structure of IBS samples slightly increased. With the increase of wt % of the bioceramic component, the % strain required to break the network structure of IBS decreased. Therefore, in order to maintain the structural integrity of IBS samples, 0.1% strain was applied for frequency, temperature, and time sweep measurements. Figure 6 shows the frequency-dependent rheological results performed in the linear viscoelastic region under 0.1% strain. 

In the measured frequency range, all of the IBS samples had a higher storage modulus than the loss modulus, confirming the gelation and stabilization of their structure after setting at 37 °C [24,40]. The setting takes place in two stages. In the first stage, hardening occurs either by the hydration of the salts in the powder component or by a chelate reaction between MC and citric acid. At this time, the polymeric component and bioceramic components also have hydrogen bonds and ionic interactions. In the second stage of cement setting, the hardening occurs via the transformation of the bioceramic component to hydroxyapatite [9]. 

**Figure 7:**
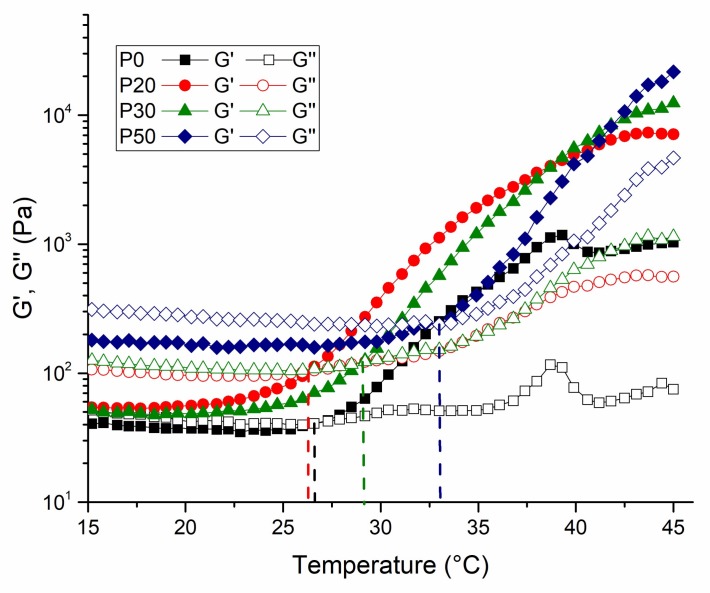
Temperature-dependent variation of G′ and G″ of IBS samples at 0.1% strain and 10 rad/s angular frequency (results expressed as mean ± standard error, n = 3).

The rheological properties of the IBS samples were evaluated by the oscillatory rheometer as a function of temperature and time. Temperature and time sweep measurements were taken to examine the impact of bioceramic powder phase on the gelation and hardening mechanism. Figure 7 shows the temperature-dependent changes of G′ and G″ of IBS samples. 

**Figure 8:**
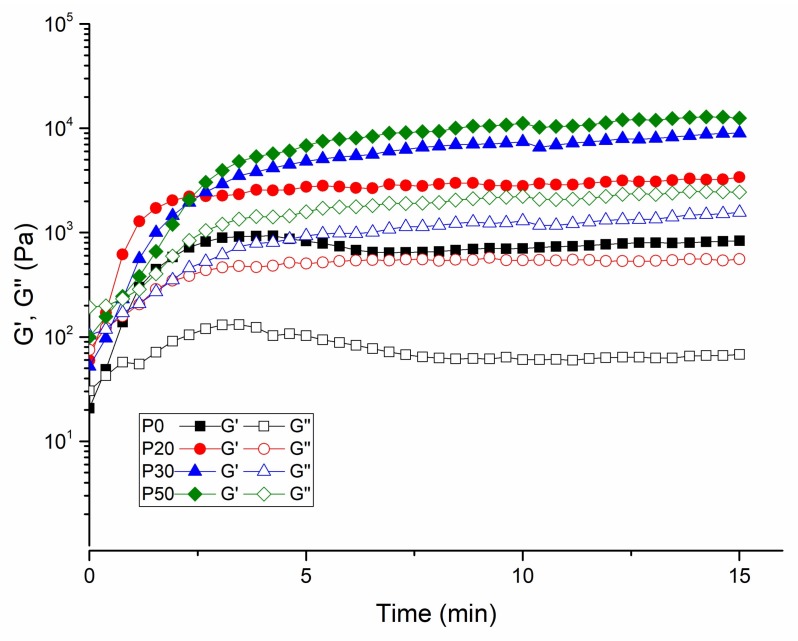
Time-dependent G′ and G″ of IBS samples measured at 37 °C, 0.1% strain, and 10 rad/s frequency (results expressed as mean ± standard error, n = 3).

The exponential increase of storage modulus with temperature implies the phase transition of the samples. The sol-gel transition temperature of the IBS samples shifted to a higher value with the addition of 30 and 50% of bioceramic mixture. The shifting of gelation temperature might be due to the change of the intra-molecular and inter-molecular interactions of MC chains [7]. The strong hydrogen bond between MC chains and CPC causes a change in the temperature sensitivity of the MC chains [49]. Figure 8 shows the gelation time at 37 °C.

Similar to the temperature sweep test, the exponential increase of the storage modulus with time at 37 °C suggests the hardening of IBS samples. The plateau point indicates the curing of the polymeric chains. The duration of curing decreases effectively with the increase of wt % of the bioceramic component [50,51,52].

**Figure 9:**
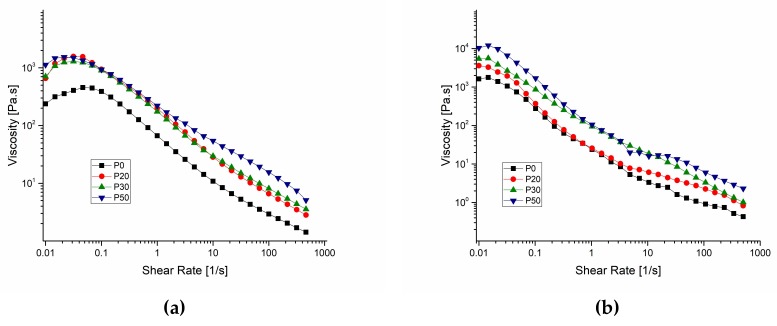
Shear-rate dependent variation of viscosity for IBS samples measured at (**a**) 25 °C and (**b**) 37 °C, respectively (results expressed as mean ± standard error, n = 3).

Figure 9 shows the shear-rate dependent variation of viscosity for IBS samples both at 25 °C and 37 °C. 

IBS samples were tested for their viscosity variation against change in the shear rate at 25 °C and 37 °C to observe whether IBS samples keep their injectability with respect to the increase of wt % of the bioceramic component. The results reveal that both at 25 °C and 37 °C, all of the samples had shear thinning properties and P0 had a considerably lower viscosity when compared to P20, P30 and P50 [24,41]. When the wt % of bioceramic component increased, viscosity also increased. This is due to the increase of resistance to flow as the number of the cement particles per unit volume increases [49]. 

### 3.5. pH Change

**Figure 10:**
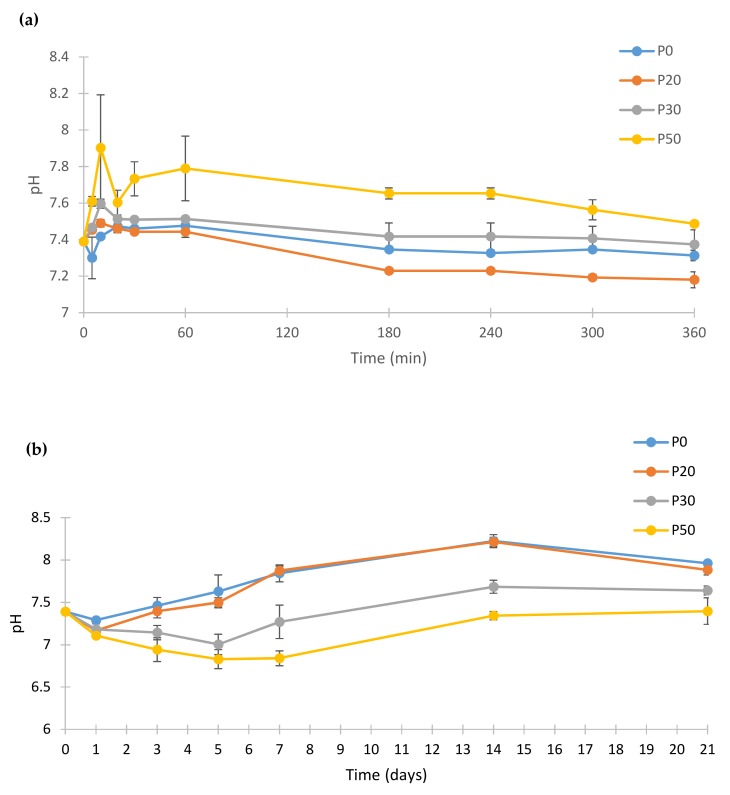
pH change of the PBS (phosphate buffer saline) that IBS samples incubated at 37 °C. (**a**) pH changes of IBS samples until 360 min and (**b**) pH changes of IBS samples until 21st day (results expressed as mean ± standard error, n = 3).

Figure 10 shows the pH change of the PBS after incubation of IBS samples at 37 °C. 

The pH profile of biomaterials in PBS is an important indicator of some of their possible biological responses. The pH response of the samples were measured for 21 days to monitor the pH changes after setting of IBS samples during the dissolution and re-precipitation. IBS samples had pH values between 7.89 and 7.39 at the end of day 21. Hence, it can be concluded that the prepared IBS samples may not cause any inflammatory reaction under biological conditions due to acidity. The pH of P50 samples was found to be higher than the other IBS samples at the beginning of incubation. P50 samples had the highest pH value until 60 min after which pH was gradually decreased. For P0, P20 and P30 IBS samples, the pH value reached a plateau after 3 h until the end of the first day. After day 1, pH values of P0, P20, and P30 started to increase slightly. The increase of pH after the 1st day of incubation was possibly due to the dissolution and transformation of TTCP into HA as indicated by Yokoyama et al. [41]. For P30 and P50 samples, the reduction of pH value until day 5 results in PO_4_^3−^ consumption which leads to formation of an apatite-like phase [49]. 

### 3.6. In Vitro Degradation

**Figure 11:**
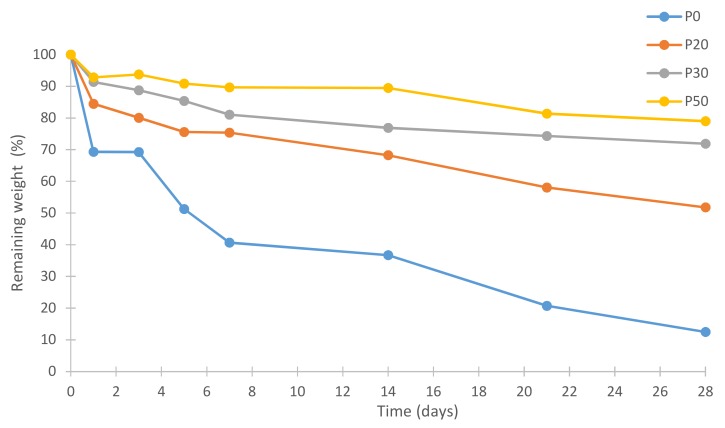
Remaining weights of IBS samples in PBS at 37 °C (results expressed as mean ± standard error, n = 3).

Figure 11 shows % remaining weights of all IBS samples.

The in vitro degradation behavior of IBS samples was investigated by the measurement of % weight loss in PBS at 37 °C after the setting of the cement phase. After one week, P0 samples lost 60% of their weight due to the erosion of MC as Gupta et al. [53] and Tate et al. [54] reported. When the wt % of bioceramic powder component increased, the weight loss decreased. In vitro degradation studies were conducted without utilizing any enzymes. Therefore, a faster degradation rate of the IBS system is expected under in vivo conditions [55,56,57]. Therefore, the degradation rate of the IBS system may be further decreased with use of additives.

### 3.7. XRD Analysis

**Figure 12:**
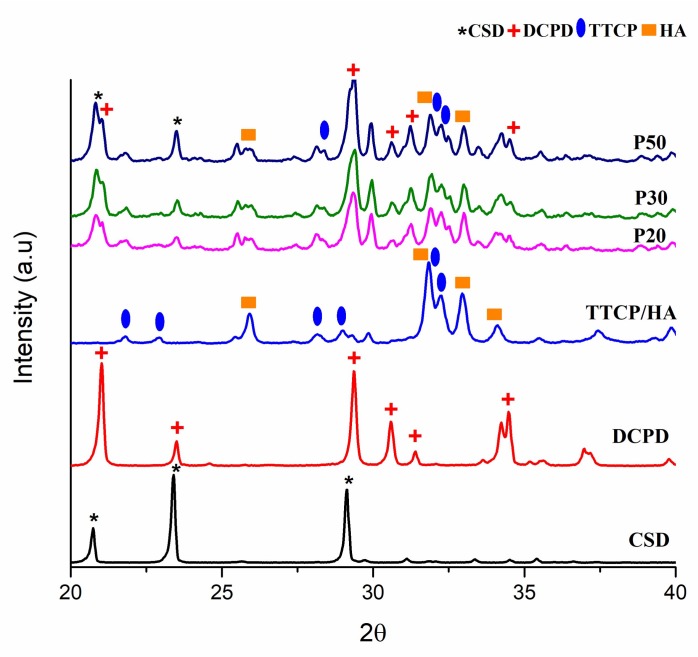
XRD analysis results of P20, P30 and P50 samples after setting.

After the cement phase of the IBS samples was allowed to set, XRD analysis was conducted. Figure 12 shows the XRD patterns of the IBS samples. 

**Figure 13:**
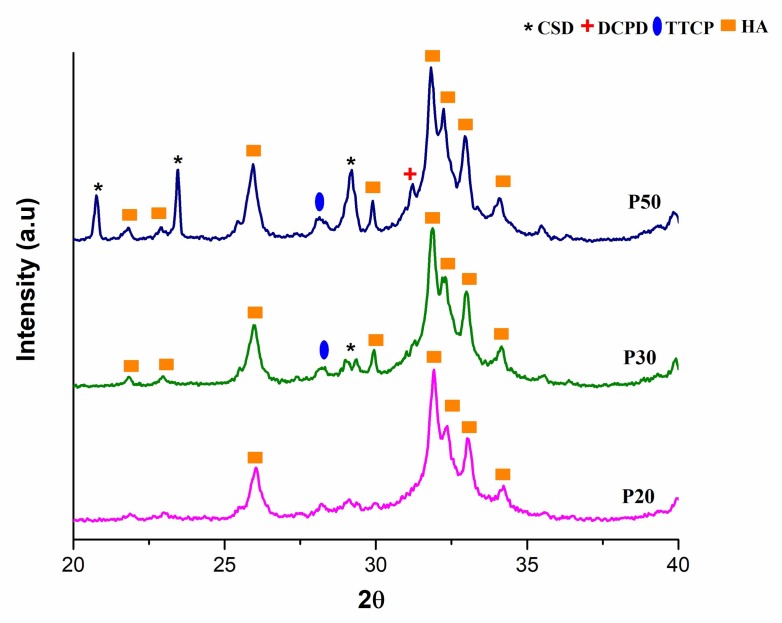
XRD analysis results of P20, P30 and P50 samples incubated in PBS at 37 °C for 14 days.

As a result of these analyses, the peaks of powder components, TTCP, HA, DCPD, and CSD were observed from the XRD spectrum of P20, P30, and P50. The peak intensity of each IBS sample was increased as the wt % of the bioceramic powder increased. As Thai and Lee [9] concluded, initial XRD data did not reveal the setting mechanism of P20, P30, and P50 samples. Therefore, the XRD analysis was also performed after PBS studies to interpret the setting mechanism and mechanical behavior after incubation for 14 days. Figure 13 shows the XRD analysis after the incubation of IBS samples in PBS at 37 °C.

The deposition of an apatite layer on bone substitutes in the biological environment is an essential phenomenon as it indicates the osseointegration ability of implants [56]. The XRD results of the incubated IBS samples showed HA peaks which indicated the formation of an apatite layer on the samples. CSD peaks were also observed for P50 which is possibly due to a higher wt % of CSD present in P50 samples. 

### 3.8. Compressive Strength Measurements

**Figure 14:**
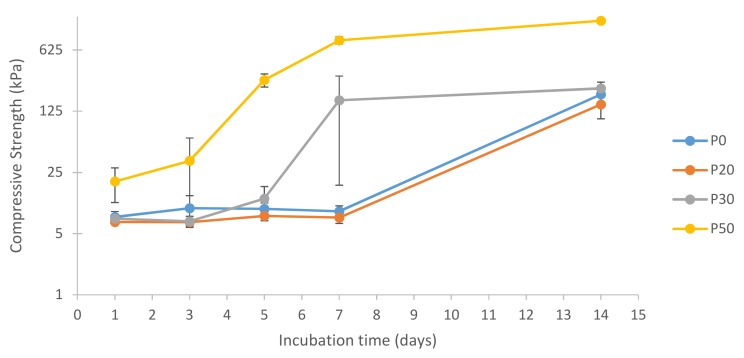
Log compressive strength of IBS samples vs. incubation time in 100% humidity at 37 °C (results expressed as mean ± standard error, n = 6).

Figure 14 shows the compressive strength results of IBS samples in 100% humidity at 37 °C. 

Figure 14 shows the compressive strength values of the samples after incubation in 100% humidity at 37 °C for 14 days. According to the results, P0 and P20 had almost the same compressive strength values. 

Until day 7, the compressive strength of P30 samples had a similar trend with P0 and P20. However, interestingly, P30 had a significant increase in compressive strength on day 7. This increase was correlated with XRD results which indicated the phase transformation of TTCP into HA [41]. P50 samples had a much earlier rise in compressive strength than P30 samples; however, both P30 and P50 reached a plateau on day 7 indicating the completion of their phase transformation into HA. On day 14, P50 samples had approximately 7 times higher compressive strength when compared to P0, P20, and P30 samples. The compressive strength of cancellous bone varies between 0.22 to 10.44 MPa [4,58,59]. Compared to human cancellous bone, the compressive strength of IBS was found to be lower. One way to improve the mechanical properties of IBS is to increase the wt % of the bioceramic component. In this study, wt % of the bioceramic component could not be increased further due to the inability to inject IBS with higher wt % of the bioceramic component. The mechanical properties of IBS may be improved with the addition of carbon-based nanomaterials, such as carbon nanotubes and graphene oxide [60,61,62]. 

## 4. Conclusions

In this study, novel IBS were prepared by incorporation of different wt % of CaP/CS-based bioceramic powder into an MC-based solution. The rheological studies revealed that all of the samples had shear thinning properties; therefore, they had a high degree of injectability. This study showed that the incorporation of the bioceramic powder into MC-based polymeric matrices may improve the rheological, mechanical and degradation properties of IBS. In the future, it would be worthwhile to analyze the biocompatibility and biological responses of the developed IBS. Overall, the prepared IBS samples are promising candidates for the treatment of bone defects for non-load bearing applications. 

## Figures and Tables

**Table 1:** Composition of the injectable bone substitutes (IBS) samples.

Abbreviation	Gelatin (wt %)	SC (wt %)	MC (wt %)	Bioceramic Powder Component (wt %)
P0	2.5	3	8	0
P20	2.5	3	8	20
P30	2.5	3	8	30
P50	2.5	3	8	50

## Supplementary Materials

The following are available online at http://www.mdpi.com/1996-1944/11/4/604/s1, Figure S1: TTCP phase identified by XRD with JCPDS No. 25-1137, No. 09-0432 and No. 09-0080 files, Figure S2: Extrusion videos of IBS samples through syringe with 18-gauge needle. in PBS at 37 °C (**a**) extrusion of P0, (**b**) extrusion of P20, (**c**) extrusion of P30 and (**d**) extrusion of P50., Figure S3: Injectability of P0, P20, P30 and P50 samples.

## References

[1] Brown， E., Chow L.C. (1983). A New Calcium Phosphate, Setting Cement. J. Dent..

[2] Low K.L., Tan S.H., Zein S.H.S., Roether J.A., Mouriño V., Boccaccini A.R. (2010). Calcium phosphate-based composites as injectable bone substitute materials. J. Biomed. Mater. Res. Part B Appl. Biomater..

[3] Ginebra M.P., Espanol M., Montufar E.B., Perez R.A., Mestres G. (2010). New processing approaches in calcium phosphate cements and their applications in regenerative medicine. Acta Biomater..

[4] O’Neill R., McCarthy H.O., Montufar E.B., Ginebra M.P., Wilson D.I., Lennon A., Dunne N. (2017). Critical review: Injectability of calcium phosphate pastes and cements. Acta Biomater..

[5] Lewis G. (2006). Injectable bone cements for use in vertebroplasty and kyphoplasty: state-of-the-art review. J. Biomed. Mater. Res. B Appl. Biomater..

[6] Habraken W., Habibovic P., Epple M., Bohner M. (2016). Calcium phosphates in biomedical applications: Materials for the future?. Mater. Today.

[7] Chow L.C. (2009). Next generation calcium phosphate-based biomaterials. Dent. Mater. J..

[8] Barrère F., van Blitterswijk C.A., de Groot K. (2006). Bone regeneration: Molecular and cellular interactions with calcium phosphate ceramics. Int. J. Nanomed..

[9] Thai V.V., Lee B.T. (2010). Fabrication of calcium phosphate-calcium sulfate injectable bone substitute using hydroxy-propyl-methyl-cellulose and citric acid. J. Mater. Sci. Mater. Med..

[10] Rangabhatla A.S.L., Tantishaiyakul V., Oungbho K., Boonrat O. (2016). Fabrication of pluronic and methylcellulose for etidronate delivery and their application for osteogenesis. Int. J. Pharm..

[11] Chen Z., Zhang X., Kang L., Xu F., Wang Z., Cui F.-Z., Guo Z. (2015). Recent progress in injectable bone repair materials research. Front. Mater. Sci..

[12] Perez R.A., Shin S.-H., Han C.-M., Kim H.-W. (2015). Bioactive injectables based on calcium phosphates for hard tissues: A recent update. Tissue Eng. Regen. Med..

[13] Kondiah P.J., Choonara Y.E., Kondiah P.P.D., Marimuthu T., Kumar P., Du Toit L.C., Pillay V. (2016). A review of injectable polymeric hydrogel systems for application in bone tissue engineering. Molecules.

[14] Wang L., Zhang C., Li C., Weir M.D., Wang P., Reynolds M.A., Zhao L., Xu H.H.K. (2016). Injectable calcium phosphate with hydrogel fibers encapsulating induced pluripotent, dental pulp and bone marrow stem cells for bone repair. Mater. Sci. Eng. C.

[15] Priya M.V., Sivshanmugam A., Boccaccini A.R., Goudouri O.M., Sun W., Hwang N., Deepthi S., Nair S.V., Jayakumar R. (2016). Injectable osteogenic and angiogenic nanocomposite hydrogels for irregular bone defects Injectable osteogenic and angiogenic nanocomposite hydrogels for irregular bone defects. Biomed. Mater..

[16] Shimokawa K., Saegusa K., Ishii F. (2009). Rheological properties of reversible thermo-setting in situ gelling solutions with the methylcellulose-polyethylene glycol-citric acid ternary system (2): Effects of various water-soluble polymers and salts on the gelling temperature. Colloid Surf. B Biointerfaces.

[17] Basnett P., Knowles J.C., Pishbin F., Smith C., Keshavarz T., Boccaccini A.R., Roy I. (2012). Novel biodegradable and biocompatible poly(3-hydroxyoctanoate)/bacterial cellulose composites. Adv. Eng. Mater..

[18] Perale G., Rossi F., Santoro M., Peviani M., Papa S., Llupi D., Torriani P., Micotti E., Previdi S., Cervo L. (2012). Multiple drug delivery hydrogel system for spinal cord injury repair strategies. J. Control. Release.

[19] Bain M.K., Maity D., Bhowmick B., Mondal D., Mollick M.M.R., Sarkar G., Bhowmik M., Rana D., Chattopadhyay D. (2013). Effect of PEG-salt mixture on the gelation temperature and morphology of MC gel for sustained delivery of drug. Carbohydr. Polym..

[20] Jeong N., Park J., Yoo K., Kim W., Kim D.H., Yoon S.Y. (2016). Preparation, characterization, and in-vitro performance of novel injectable silanized-hydroxypropyl methylcellulose/phase-transformed calcium phosphate composite bone cements. Curr. Appl. Phys..

[21] Ghanaati S., Barbeck M., Hilbig U., Hoffmann C., Unger R.E., Sader R.A., Peters F., Kirkpatrick C.J. (2011). An injectable bone substitute composed of beta-tricalcium phosphate granules, methylcellulose and hyaluronic acid inhibits connective tissue influx into its implantation bed in vivo. Acta Biomater..

[22] Krause M., Oheim R., Catala-Lehnen P., Pestka J.M., Hoffmann C., Huebner W., Peters F., Barvencik F., Amling M. (2014). Metaphyseal bone formation induced by a new injectable beta-TCP-based bone substitute: A controlled study in rabbits. J. Biomater. Appl..

[23] Patenaude M., Hoare T. (2012). Injectable, mixed natural-synthetic polymer hydrogels with modular properties. Biomacromolecules.

[24] Liu Z., Yao P. (2015). Injectable thermo-responsive hydrogel composed of xanthan gum and methylcellulose double networks with shear-thinning property. Carbohydr. Polym..

[25] Félix Lanao R.P., Sariibrahimoglu K., Wang H., Wolke J.G.C., Jansen J.A., Leeuwenburgh S.C.G. (2014). Accelerated calcium phosphate cement degradation due to incorporation of glucono-delta-lactone microparticles. Tissue Eng. Part A.

[26] Dessì M., Alvarez-Perez M.A., De Santis R., Ginebra M.P., Planell J.A., Ambrosio L. (2014). Bioactivation of calcium deficient hydroxyapatite with foamed gelatin gel. A new injectable self-setting bone analogue. J. Mater. Sci. Mater. Med..

[27] Utech S., Boccaccini A.R. (2016). A review of hydrogel-based composites for biomedical applications: enhancement of hydrogel properties by addition of rigid inorganic fillers. J. Mater. Sci..

[28] Bongio M., Nejadnik M.R., Kasper F.K., Mikos A.G., Jansen J.A., Leeuwenburgh S.C.G., van den Beucken J.J.J.P. (2013). Development of an in vitro confinement test to predict the clinical handling of polymer-based injectable bone substitutes. Polym. Test..

[29] Nishinari K., Hofmann K.E., Kohyama K., Moritaka H., Nishinari N., Watase M. (1993). Polysaccharide-protein interaction: A rheological study of the gel-sol transition of a gelatin-methylcellulose-water system. Biorheology.

[30] Demitri C., Del Sole R., Scalera F., Sannino A., Vasapollo G., Maffezzoli A., Ambrosio L., Nicolais L. (2008). Novel superabsorbent cellulose-based hydrogels crosslinked with citric acid. J. Appl. Polym. Sci..

[31] Habraken W.J.E.M., Jonge L.T., De Wolke J.G.C., Yubao L., Mikos A.G., Jansen J.A. (2008). Introduction of gelatin microspheres into an injectable calcium phosphate cement. J. Biomed. Mater. Res. A.

[32] Sanmartín-Masiá E., Poveda-Reyes S., Gallego Ferrer G. (2017). Extracellular matrix–inspired gelatin/hyaluronic acid injectable hydrogels. Int. J. Polym. Mater. Polym. Biomater..

[33] Tang Y., Wang X., Li Y., Lei M., Du Y., Kennedy J.F., Knill C.J. (2010). Production and characterisation of novel injectable chitosan/methylcellulose/salt blend hydrogels with potential application as tissue engineering scaffolds. Carbohydr. Polym..

[34] Sadiasa A., Sarkar S.K., Franco R.A., Min Y.K., Lee B.T. (2013). Bioactive glass incorporation in calcium phosphate cement-based injectable bone substitute for improved in vitro biocompatibility and in vivo bone regeneration. J. Biomater. Appl..

[35] Hempel U., Reinstorf A., Poppe M., Fischer U., Gelinsky M., Pompe W., Wenzel K.W. (2004). Proliferation and Differentiation of Osteoblasts on Biocement D Modified with Collagen Type I and Citric Acid. J. Biomed. Mater. Res. Part B Appl. Biomater..

[36] Wang X., Ye J., Wang H. (2005). Effects of Additives on the Rheological Properties and Injectability of a Calcium Phosphate Bone Substitute Material. J. Biomed. Mater. Res. Part B Appl. Polym..

[37] Guo D., Xu K., Han Y. (2005). Influence of cooling modes on purity of solid-state synthesized tetracalcium phosphate. Mater. Sci. Eng. B Solid-State Mater. Adv. Technol..

[38] Song H.Y., Rahman A.H.M.E., Lee B.T. (2009). Fabrication of calcium phosphate-calcium sulfate injectable bone substitute using chitosan and citric acid. J. Mater. Sci. Mater. Med..

[39] Alves H.L.R., dos Santos L.A., Bergmann C.P. (2008). Injectability evaluation of tricalcium phosphate bone cement. J. Mater. Sci. Mater. Med..

[40] Fan R.R., Deng X.H., Zhou L.X., Gao X., Fan M., Wang Y.L., Guo G. (2014). Injectable thermosensitive hydrogel composite with surface-functionalized calcium phosphate as raw materials. Int. J. Nanomed..

[41] Yokoyama A., Yamamoto S., Kawasaki T., Kohgo T., Nakasu M. (2002). Development of calcium phosphate cement using chitosan and citric acid for bone substitute materials. Biomaterials.

[42] Huang Z., Feng Q., Yu B., Li S. (2011). Biomimetic properties of an injectable chitosan / nano-hydroxyapatite / collagen composite. Mater. Sci. Eng. C.

[43] Radwan M.M., Abd El-Hamid H.K., Nagi S.M. (2016). Synthesis, properties and hydration characteristics of novel nano-size mineral trioxide and tetracalcium phosphate for dental applications. Orient. J. Chem..

[44] Jayasree R., Kumar T.S., Kavya K.P.S., Nankar P.R., Mukesh D. (2015). Self Setting Bone Cement Formulations Based on Egg shell Derived TetraCalcium Phosphate BioCeramics. Bioceram. Dev. Appl..

[45] Liao J., Duan X., Li Y., Zheng C., Yang Z., Zhou A., Zou D. (2014). Synthesis and mechanism of tetracalcium phosphate from nanocrystalline precursor. J. Nanomater..

[46] Kim H., Camata R.P., Vohra Y.K., Lacefield W.R. (2005). Control of phase composition in hydroxyapatite/tetracalcium phosphate biphasic thin coatings for biomedical applications. J. Mater. Sci. Mater. Med..

[47] Eslami H., Solati-Hashjin M., Tahriri M. (2008). Synthesis and Characterization of Hydroxyapatite Nanocrystals via Chemical Precipitation Technique. Iran. J. Pharm. Sci..

[48] Liu W., Zhang J., Rethore G., Khairoun K., Pilet P., Tancret F., Bouler J.M., Weiss P. (2014). A novel injectable, cohesive and toughened Si-HPMC (silanized-hydroxypropyl methylcellulose) composite calcium phosphate cement for bone substitution. Acta Biomater..

[49] Marefat Seyedlar R., Nodehi A., Atai M., Imani M. (2014). Gelation behavior of in situ forming gels based on HPMC and biphasic calcium phosphate nanoparticles. Carbohydr. Polym..

[50] Ghorbani M., Ai J., Nourani M.R., Azami M., Hashemi Beni B., Asadpour S., Bordbar S. (2017). Injectable natural polymer compound for tissue engineering of intervertebral disc: In vitro study. Mater. Sci. Eng. C.

[51] Arvidson S.A., Lott J.R., McAllister J.W., Zhang J., Bates F.S., Lodge T.P., Sammler R.L., Li Y., Brackhagen M. (2013). Interplay of phase separation and thermoreversible gelation in aqueous methylcellulose solutions. Macromolecules.

[52] Wu J., Liu J., Shi Y., Wan Y. (2016). Rheological, mechanical and degradable properties of injectable chitosan/silk fibroin/hydroxyapatite/glycerophosphate hydrogels. J. Mech. Behav. Biomed. Mater..

[53] Gupta D., Tator C.H., Shoichet M.S. (2006). Fast-gelling injectable blend of hyaluronan and methylcellulose for intrathecal, localized delivery to the injured spinal cord. Biomaterials.

[54] Tate M.C., Shear D.A., Hoffman S.W., Stein D.G., LaPlaca M.C. (2001). Biocompatibility of methylcellulose-based constructs designed for intracerebral gelation following experimental traumatic brain injury. Biomaterials.

[55] Ma X., Zhang L., Fan D., Xue W., Zhu C., Li X., Liu Y., Liu W., Ma P., Wang Y. (2017). Physicochemical properties and biological behavior of injectable crosslinked hydrogels composed of pullulan and recombinant human-like collagen. J. Mater. Sci..

[56] Ding Y., Tang S., Yu B., Yan Y., Li H., Wei J., Su J. (2015). In vitro degradability, bioactivity and primary cell responses to bone cements containing mesoporous magnesium–calcium silicate and calcium sulfate for bone regeneration. J. R. Soc. Interface.

[57] Qasim S.B., Husain S., Huang Y., Pogorielov M., Deineka V., Lyndin M., Rawlinson A., Rehman I.U. (2017). In-vitro and in-vivo degradation studies of freeze gelated porous chitosan composite scaffolds for tissue engineering applications. Polym. Degrad. Stab..

[58] Misch C.E., Qu Z., Bidez M.W. (1999). Mechanical properties of trabecular bone in the human mandible: Implications for dental implant treatment planning and surgical placement. J. Oral Maxillofac. Surg..

[59] Baino F. (2017). Ceramics for bone replacement. Advances in Ceramic Biomaterials.

[60] Ege D., Kamali A.R., Boccaccini A.R. (2017). Graphene Oxide/Polymer-Based Biomaterials. Adv. Eng. Mater..

[61] Newman P., Minett A., Ellis-Behnke R., Zreiqat H. (2013). Carbon nanotubes: Their potential and pitfalls for bone tissue regeneration and engineering. Nanomed. Nanotechnol. Biol. Med..

[62] Gholami F., Zein S.H.S., Gerhardt L.-C., Low K.L., Tan S.H., McPhail D.S., Grover L.M., Boccaccini A.R. (2013). Cytocompatibility, bioactivity and mechanical strength of calcium phosphate cement reinforced with multi-walled carbon nanotubes and bovine serum albumin. Ceram. Int..

