# Supplementary material for: Rheological and Mechanical Properties of Thermoresponsive Methylcellulose/Calcium Phosphate-Based Injectable Bone Substitutes
Source: Materials (Basel). 2018 Apr 14;11(4):604. doi: 10.3390/ma11040604 (PMC5951488; doi:10.3390/ma11040604)
Supplement: Supplementary file 1 [file materials-11-00604-s001.zip › Supplementary data-Calcium phosphate-based injectable bone substitutes.docx]

Rheological and Mechanical Properties of Thermoresponsive Methylcellulose/Calcium Phosphate-based Injectable Bone Substitutes

Öznur Demir Oğuz ^1^, Duygu Ege ^1*^

^1^ Institute of Biomedical Engineering, Boğaziçi University, Rasathane St., Kandilli, 34684, İstanbul Turkey; [oznur.demir@boun.edu.tr](mailto:oznur.demir@boun.edu.tr)

***** Correspondence: [duygu.ege@boun.edu.tr](mailto:duygu.ege@boun.edu.tr) ; Tel.: +90-216-516-3438

**Supplementary data**

**1. Analysis of synthesized powder**

The powder was synthesized as described in Section 2.1.2. The prepared powder was analyzed by using XRD. The quantitative analysis of synthesized powder was matched with JCPDS No. 25-1137, No. 09-0432 and No. 09-0080 of the TTCP, HA and monetite crystal structure, respectively. Figure S1 shows the quantitative analysis results of synthesized powder.


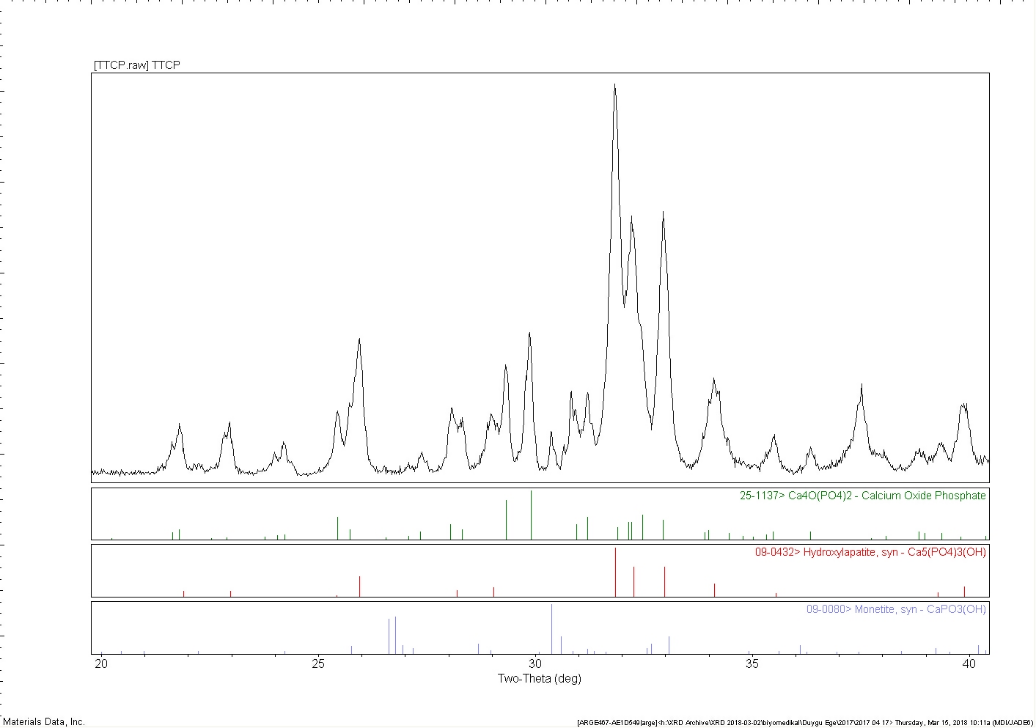


Figure S1. TTCP phase identified by XRD with JCPDS No. 25-1137, No. 09-0432 and No. 09-0080 files.

**2. Injectability of IBS samples**

After mixing the bioceramic powder and polymeric component as given ratios in Table 1, IBS samples were extruded in PBS at 37°C to examine their stability. Figure S2 shows the extrusion videos of IBS samples.


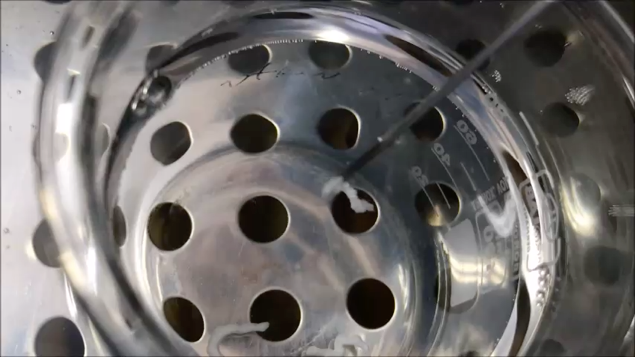


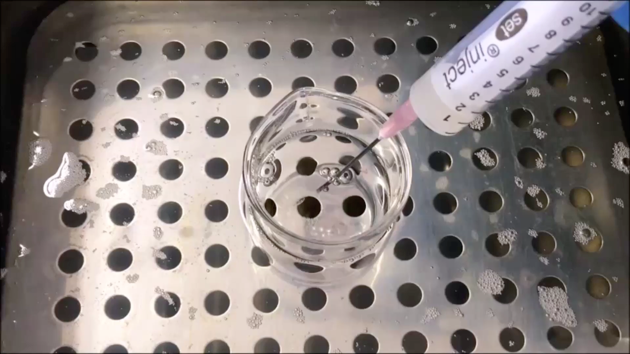


**(b)**

**(a)**

**Figure S2.** Extrusion videos of IBS samples through syringe with 18-gauge needle. in PBS at 37°C (a) extrusion of P0, (b) extrusion of P20, (c) extrusion of P30 and (d) extrusion of P50.


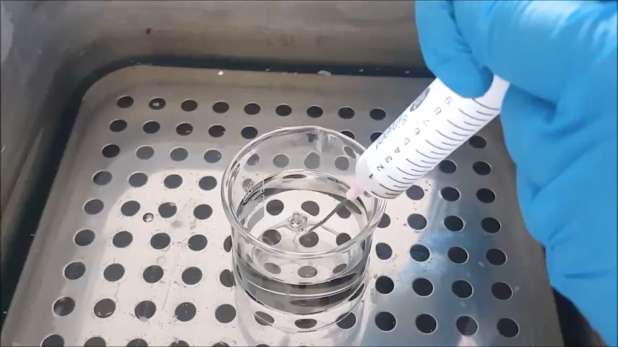

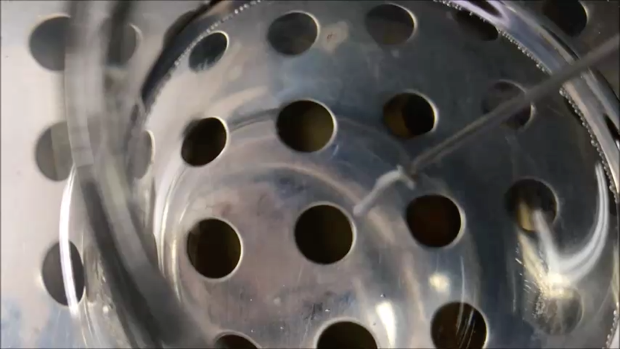


**(d)**

**(c)**

**Figure S2.** Extrution videos of IBS samples through 18-gauge needle in PBS at 37°C.

IBS samples showed cohesive stability in PBS after extrusion. wt % of the cement component differred in all IBS compositions. Figure S3 shows the degree of injectability of all IBS samples.

**Figure S3**. Injectability of P0, P20, P30 and P50 samples (results mean ± standard error, n=3).

All samples had high degree of injectablity, ranging from 94.53 to 91.73 %. As the wt % of bioceramic powder component increased, injectability was slightly decreased. Alves et al.[1] indicated that the injection process was fundamentally controlled by rheology of the paste. Therefore, a higher viscosity led to more difficulty of injection of the pastes. Despite this, all studied samples showed a high degree of injectability.

References

1. Alves, H. L. R.; dos Santos, L. A.; Bergmann, C. P. Injectability evaluation of tricalcium phosphate bone cement. *J. Mater. Sci. Mater. Med.* **2008**, *19*, 2241–2246, doi:10.1007/s10856-007-3329-6.
