# Supplementary figures and images for: Rheological and Mechanical Properties of Thermoresponsive Methylcellulose/Calcium Phosphate-Based Injectable Bone Substitutes
Source: Materials (Basel). 2018 Apr 14;11(4):604. doi: 10.3390/ma11040604 (PMC5951488; doi:10.3390/ma11040604)

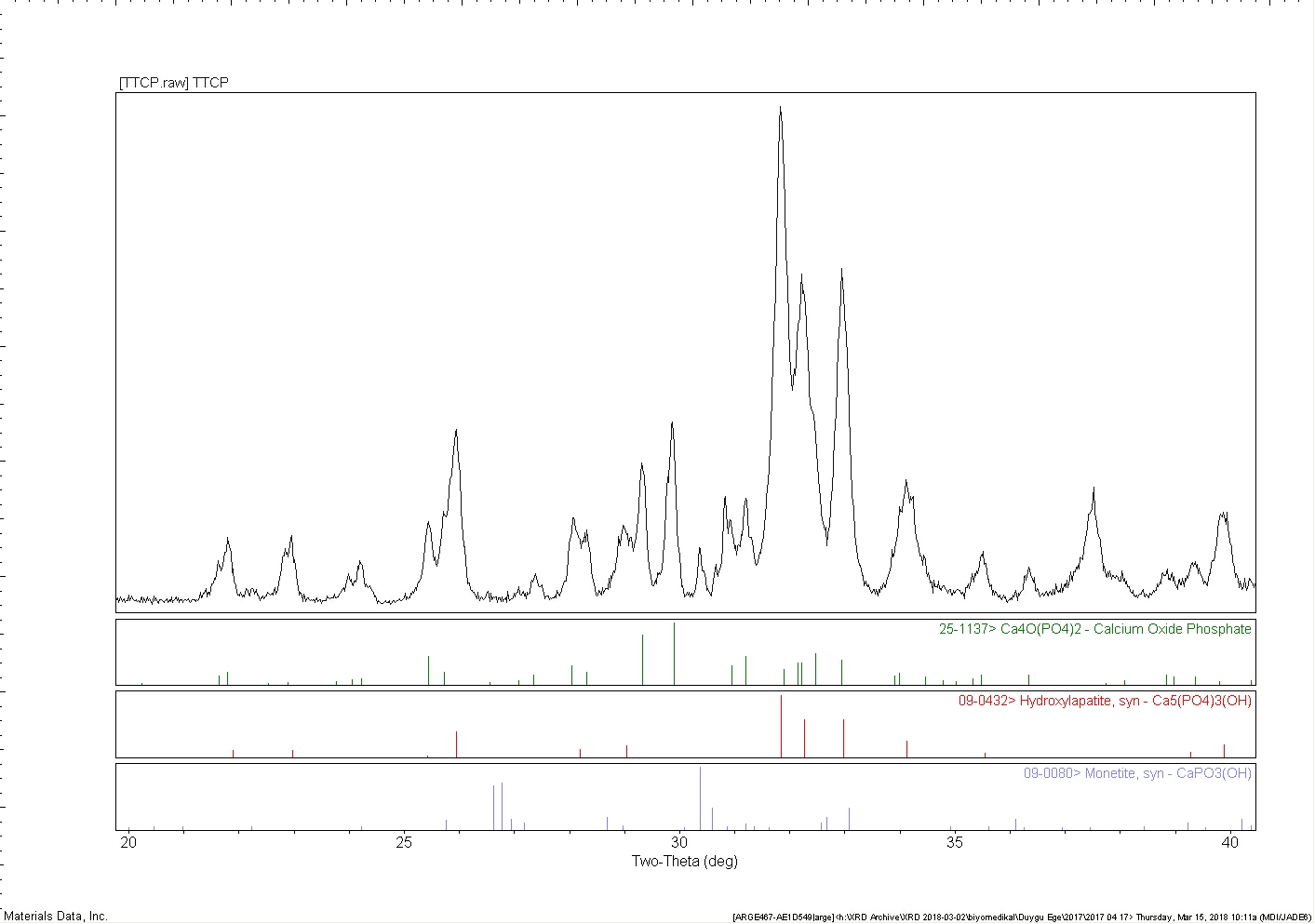

Supplement: Supplementary file 1 [file materials-11-00604-s001.zip › FigureS1.tif]

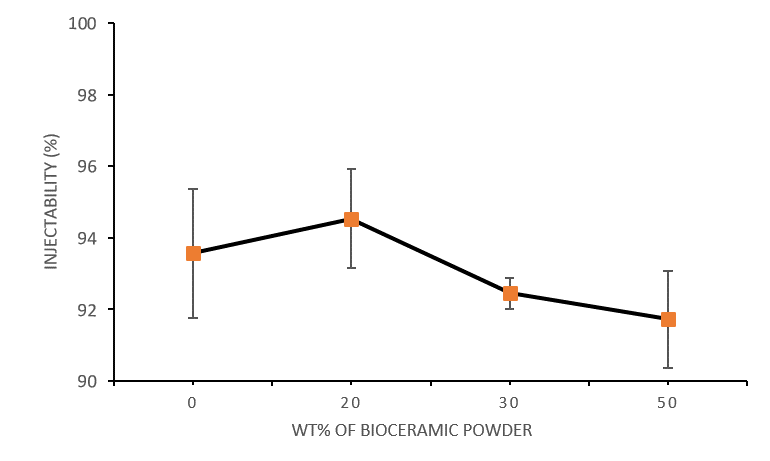

Supplement: Supplementary file 1 [file materials-11-00604-s001.zip › FigureS3.png]
